# Establishment of the MGH Postpartum Psychosis Project: MGHP3

**DOI:** 10.1371/journal.pone.0281133

**Published:** 2023-02-09

**Authors:** Lee S. Cohen, Rachel Vanderkruik, Miranda Arakelian, Taylor R. Church, Madison M. Dunk, Marlene P. Freeman

**Affiliations:** Department of Psychiatry, Ammon-Pinizzotto Center for Women’s Mental Health, Massachusetts General Hospital, Harvard Medical School, Boston, Massachusetts, United States of America; Duke University Medical Center: Duke University Hospital, UNITED STATES

## Abstract

**Objective:**

Postpartum psychosis (PP) is a severe psychiatric disorder, with incomplete consensus on definition and diagnostic criteria. The Massachusetts General Hospital Postpartum Psychosis Project (MGHP3) was established to better ascertain the phenomenology of PP in a large cohort of diverse women spanning a wide geographical range (primarily in the US), including time of onset, symptom patterns, and associated comorbidities, psychiatric diagnoses pre- and post- the episode of PP, and also to identify genomic and clinical predictors of PP. This report describes the methods of MGHP3 and provides a status update.

**Method:**

Data are collected from women who experienced PP within 6 months of childbirth and who provided this information within ten years of the study interview. Subject data are gathered during a one-time structured clinical interview conducted by phone, which includes administration of the Mini International Neuropsychiatric Interview for Psychotic Disorders Studies (Version 7.0.2), the MGHP3© Questionnaire, and other information including lifetime mental health history and use of psychiatric medications both prior to the episode of PP and during the subsequent time period prior to study interview. Subjects also provide a saliva sample to be processed for genomic analyses; a neuroimaging assessment is also conducted for a subset of participants.

**Results:**

As of July 1, 2022, 311 subjects from 44 states and 7 countries were enrolled in MGHP3. Recruitment sources include social media, online advertisements, physician referral, community outreach, and partnership with PP advocacy groups.

**Conclusions:**

The rigorous phenotyping, genetic sampling, and neuroimaging studies in this sample of women with histories of PP will contribute to better understanding of this serious illness. Findings from MGHP3 can catalyze ongoing discussions in the field regarding proper nosologic classification of PP as well as relevant treatment implications.

## Introduction

Postpartum psychosis (PP) occurs in 1–2 per 1000 women after delivery [1]. However, considering the large number of women who experience childbirth each year, a substantial number of women suffer from this disorder. PP typically manifests in the early days or weeks following delivery with the onset of psychotic symptoms including delusional thinking, paranoia, and hallucinations; symptoms most often occur in the context of an episode of mania or depression [2]. Although uncommon, the severity of PP cannot be overstated; it is often a psychiatric emergency and requires acute attention from medical professionals with knowledge of appropriate treatment options. Women experiencing PP are at significant risk for maternal suicide, and infanticide can be a tragic complication of untreated illness [3]. Aside from mortality risk, PP is associated with increased risk of child displacement, social services involvement, and insecure infant attachment compared to healthy control groups without postpartum psychiatric illness [4]. Because PP is the index episode of a psychotic or major affective disorder for many women, it is important to identify those women who are at greatest risk for this illness [5]. Therefore, describing clinical predictors and patterns of symptom presentation is of significant clinical and public health importance.

The classification of PP is the subject of much debate, and the disorder is not recognized as a distinct diagnosis in the most recent Diagnostic and Statistical Manual (DSM) of Mental Disorders, DSM-5 [6]. While the current version of the DSM-5 allows for the diagnosis of major depressive disorder or a psychotic disorder with postpartum onset, some argue that such classification is incomplete and insufficient [7]. It has been proposed that PP be included in the DSM as a unique diagnosis based on its distinctive features (e.g., temporal onset following childbirth, cognitive disorganization or delirium in some affected patients) and that failure to appreciate such features may hinder diagnosis and treatment [7]. Furthermore, there is substantial evidence that PP may lie on the spectrum of bipolar disorders [5]. Although the presentation of PP often includes mania and/or depression, there is additional debate regarding whether PP should be classified as an episode of bipolar disorder or be considered a discrete illness [8]. For many patients without preexisting psychiatric disorders, PP is in fact the index episode of bipolar disorder, and over time a bipolar diagnosis is apparent as these patients go on to experience subsequent episodes of mania/hypomania and/or depression. A prospective study found that among women who experienced postpartum onset mania or psychosis, 32% subsequently experienced a recurrent manic, hypomanic and/or depressive episode following an index episode of PP [9]. However, for a substantial proportion of patients (43.5%), the disorder appeared to be more circumscribed, and patients did not manifest recurrent manic or psychotic illness outside of the postpartum period. This finding as well as other relevant literature suggests that while PP has significant overlap with bipolar disorder, the postpartum onset descriptor appended to a bipolar disorder diagnosis may not be sufficiently inclusive in describing PP [9, 10]. Since the long-term outcomes and risk of recurrence are still incompletely delineated, further understanding of the course of PP following the onset of an episode is of particular importance. This insight would contribute to clinical decisions regarding which patients need long-term maintenance therapy with mood stabilizers and which may be candidates to safely discontinue medications following a certain period subsequent to symptom resolution of the PP episode.

The relatively low prevalence of PP, inconsistent classification, and history of stigmatization associated with the illness makes its systematic study particularly challenging but nonetheless important. To recruit a large sample of women with histories of PP, some investigators have relied primarily on inpatient populations or populations with pre-existing diagnoses (such as bipolar disorder) for recruitment [8]. The lack of consistency in PP diagnostic criteria and naming conventions, as well as the absence of an International Classification of Diseases (ICD) code used for categorization and billing purposes, makes the conduct of system-wide analyses of patient records particularly challenging. Such heterogeneity of definitions also contributes to the challenge of collaborative research on PP. For example, some investigators include patients with isolated mania (even in the absence of psychotic symptoms) under the diagnostic umbrella of PP [8], and the definition of the postpartum window varies significantly across studies (from within 4 weeks to within 6 months) [11, 12].

In response to this identified need to more adequately describe and classify PP, research has sought to further elucidate the phenomenology, symptomatology, and clinical status of those who suffer from PP. Common clinical features identified include affective symptoms (mania and/or depression), visual or auditory hallucinations, disorganized thoughts, ideas of reference, and delusional ideas [2]. A prospective cohort study performed in the Netherlands assessed the symptoms and phenotypes of 130 inpatient participants with PP and classified them into mutually exclusive, homogeneous subgroups (i.e., depressive, manic, and atypical) [13]. Given the observed familial clustering of PP/mania, previous research has also examined genetic contributions to PP; Jones et al. [14] conducted a genome-wide linkage study in families with women with bipolar disorder or schizoaffective disorder who experienced PP and observed linkage signals on specific chromosomes, suggesting genetic risk factors. Although there have been few treatment studies, the rate of response to lithium in the available data further suggest a bipolar diathesis [15]. Risk factors for PP include a personal or family history of PP, a history of bipolar disorder or psychotic illness prior to delivery, a family history of bipolar disorder, and cessation of anti-manic medication during pregnancy [12]. Other associated factors include fluctuations in reproductive hormones [16], loss of sleep [17], environmental stressors, autoimmune dysregulation [18], and thyroid dysregulation [19–22].

Despite these known risk factors, there are still significant gaps in knowledge and challenges in identifying patients at risk for this illness. Given the ongoing debate about classification of PP, further research is needed to better understand the etiology and phenomenology of this illness. This report describes the methods of The Massachusetts General Hospital Postpartum Psychosis Project (MGHP3), which aims to address the above-mentioned gaps in knowledge by enrolling a large and geographically diverse sample of women with histories of PP. This report describes the design, rationale, and assessments used to study the sample enrolled to-date in this ongoing study.

## Methods

### Study aims

The overarching aims of MGHP3 are the following: 1) to describe the phenomenology of PP with respect to spectrum of psychotic symptoms, co-occurring psychiatric symptoms, timing of symptom onset, and associated comorbidities, and 2) to contribute to knowledge of clinical and genomic predictors of PP. An ancillary study of MGHP3 also includes neuroimaging assessment of subjects with histories of PP in order to ascertain patterns of neural circuitry seen in women with histories of PP compared to those without histories of postpartum psychotic illness. Assessment includes structural and functional (task-based and resting-state) MRI scans to explore whether women with a PP history show abnormalities in brain structure or function congruent with previous findings in other psychiatric disorders, such as cortical thinning [23] or amygdala overactivity [24].

### Inclusion and exclusion criteria

Women are included in the study if they meet the following inclusion criteria: 1) age 18 years or older, and 2) have experienced a psychotic episode or an episode of mania with or without psychotic features within 6 months of a live birth, still birth, or intrauterine fetal demise in the past 10 years. We chose to include women who experienced a psychotic episode or an episode of mania without psychotic features in an effort to include all relevant cases of PP given the ongoing debate in the field about the nosology of PP. Subjects are excluded if they 1) have a history of a primary psychotic disorder in the absence of mood symptoms prior to the postpartum period, including schizophrenia, schizoaffective disorder, delusional disorder, schizophreniform disorder, psychotic disorder due to another medical condition, substance/medication-induced psychotic disorder, or unspecified schizophrenia spectrum and other psychotic disorder, 2) are not fluent in English, or 3) report active substance abuse within the postpartum time frame, with the exception of intermittent marijuana use.

### Subject recruitment

As all components of this study are conducted by phone or mail, recruitment for this study extends across the United States as well as internationally. Primary recruitment efforts include outreach through a web presence (i.e., womensmentalhealth.org), social media, physician referral, advocacy-based digital networks, and within Massachusetts General Hospital’s (MGH) Psychiatry Department. The Postpartum Psychosis Project website (MGHP3.org) includes resources describing PP symptoms, existing research, clinical vignettes from women who have experienced episodes of PP, and information regarding support groups. The MGHP3.org website also includes a consultation service for providers across the country who solicit guidance from MGH perinatal psychiatrists regarding the treatment of patients with PP, creating a valuable platform for patients and research participants, as well as their providers, families, and community. Engagement with community advocacy groups, social media-based support groups, and other digital networks facilitates recruitment of a substantial international sample of research subjects.

### Enrollment and interview

Potential study participants are screened for inclusion over the phone by a trained research coordinator, and verbal consent for participation is obtained and documented for eligible individuals. Following screening and consent, a one-time subject interview is conducted by telephone, lasting approximately 90 minutes. All data, including documentation of verbal consent, are stored in Research Electronic Data Capture (REDCap) [25, 26], and study procedures are conducted in accordance with the Declaration of Helsinki and with approval of the Mass General Brigham Human Research Committee Institutional Review Board. This structured clinical interview includes the *Childhood Trauma Questionnaire and Recent Trauma Events Scale* [27], a tool assessing traumatic experiences up to age 18 and within three years prior to the pregnancy of interest, and the *Massachusetts General Hospital Postpartum Psychosis Project Questionnaire*© (MGHP3-Q), a standardized questionnaire created for this study to characterize demographics, medical and psychiatric history, family history, psychiatric diagnoses (historic and current), and medical and psychiatric treatment during the pre-pregnancy, pregnancy, and postpartum periods of interest. The MGHP3-Q also captures participants’ narrative descriptions of their experience with PP (see S1 Appendix).

For rigorous assessment of psychiatric history and diagnoses, a research coordinator also conducts the Mini International Neuropsychiatric Interview for Psychotic Disorders Studies (MINI-PDS), a validated, structured diagnostic tool to assess psychiatric disorders including specific psychotic disorders in accordance with DSM-5 symptom criteria [6]. The MINI-PDS is used to establish baseline diagnoses for each participant and to ascertain whether participants experienced a psychotic, depressive, manic, or mixed episode during the postpartum period. A subsection within the MINI-PDS, Module K, assesses the presence of a range of psychotic symptoms allowing for a psychotic episode diagnosis. To accurately assess postpartum symptoms, pre-existing psychiatric diagnoses, and psychiatric symptoms since the postpartum episode, the periods assessed in the MINI-PDS for specific psychiatric disorders are modified to assess lifetime pre-pregnancy, the pregnancy of interest, and two postpartum periods: 1) from childbirth to 6 months postpartum and 2) from 6-months postpartum to the date of the interview. For participants who have experienced more than one episode of PP, the MINI-PDS is administered with respect to each pregnancy and postpartum period proximate to each episode of PP. Patient medical records are requested and obtained to further clarify diagnosis if necessary, following the MINI-PDS and consultation with the study principal investigator.

Outcomes of interest for this study include timing of PP episode onset and duration, PP symptoms, treatment received, and underlying psychiatric comorbidities. The “time of symptom onset” for a PP episode is determined by patient self-report and identifies the point at which the participant codes positive for symptoms included in the MINI-PDS Module K. “Time back to wellness” is defined as the length of time since onset of psychotic symptoms needed for the patient to get well from psychiatric symptoms such that she feels back to her baseline. This is gathered in the MGHP3-Q by participant self-report. “Length of psychotic episode” is captured by participant self-report on both the MGHP3-Q as well as the MINI-PDS Module K (which is used to identify the time at which the patient no longer meets criteria for a psychotic disorder on the MINI-PDS since time of symptom onset, measured in weeks). A participant’s symptoms are considered “ongoing” if psychiatric symptoms including but not limited to psychotic symptoms continue for the patient without a period of psychiatric wellness since symptom onset in the postpartum period. Therefore “ongoing” symptoms refer to any psychiatric symptoms, not necessarily psychotic symptoms.

Symptom severity that meets threshold criteria for psychotic diagnoses are classified according to the MINI-PDS using Diagnostic Algorithms I and II for Psychotic Disorders and Diagnostic Algorithm III for Mood Disorders. Subjects are considered to have had a psychotic episode if they code positive for a primary psychotic disorder or mood disorder with psychotic features on Module K of the MINI-PDS (Psychotic Disorders and Mood Disorders with Psychotic Features) during the 6-month postpartum period for which no organic or substance-induced cause is identified. Hospitalization refers to patient admission to an inpatient unit and does not include visits to the emergency room for which a patient is not admitted.

### DNA collection and genomic analyses

Following the completion of the interview, DNA Genotek Oragene OGR-DISCOVER 600 saliva collection kits, self-collection instructions, and paid return envelopes are mailed to subjects. DNA samples are returned to MGH and processed for genome-wide analysis and genotype quality control using the Multi-Ethnic Genotyping Array (MEGA) SNP chip at the Massachusetts General Hospital Core Laboratory.

### Neuroimaging assessment

A neuroimaging assessment is ongoing for a subgroup of MGHP3 participants living in the US and a comparison group of healthy women who have given birth but have not experienced symptoms of a psychiatric disorder, including PP. A future report will describe in greater detail the methodology for the neuroimaging assessment in this cohort of women. In short, this assessment includes well-validated structural and functional (including task-based [28–30] and resting-state [31–33] functional magnetic resonance imaging (fMRI)) scans. The fMRI data will be used to measure neural responses to emotionally salient stimuli and the connectivity of brain networks [34] involved in affective responses, salience detection and sensorimotor function [35]. This arm of MGHP3 explores whether women with a history of PP show abnormalities in brain structure or function that are similar to those previously observed in psychotic disorders, mood disorders, or both [24, 35–37].

## Study update and discussion

As of July 1, 2022, 311 subjects have been enrolled in MGHP3. Data collection began in 2018 and currently has a goal of enrolling 400 patients into the index cohort. Primary recruitment sources include web presence, social media, digital advertising partnerships, the Postpartum Psychosis Project website (MGHP3.org), and Massachusetts General Hospital (MGH) Psychiatry Department physician referral. The study has recruited from 44 states, Washington D.C., and 7 different countries to-date, and the distribution of participants captures the experience of patients across various healthcare systems, from urban specialized medical centers to rural emergency care settings.

Through continued enrollment of the study sample and the pairing of genomic and clinical data collection, we aim to assess the degree to which PP risk reflects higher polygenic loading for schizophrenia or bipolar disorder versus a novel genetic signature. Because the Psychiatric Genomics Consortium [38] polygenic risk analyses currently include primarily subjects of European ancestry, we will limit genome-wide polygenic risk analyses to that population; the Psychiatric Genomics Consortium does not yet contain summary statistics for non-white groups. MGHP3 will examine a genetic underpinning for this serious disorder and address critical questions about clinical presentation, attendant morbidity, and potential mortality of PP. Furthermore, the neuroimaging arm of MGHP3 seeks to identify whether or not women who experience psychotic symptoms within 6 months of delivery show abnormalities in brain function that are similar to those previously observed in psychotic disorders, mood disorders, or both. For this arm of the study, women without history of psychiatric illness, but who have delivered a child within the past 10 years are eligible to be enrolled as a comparison group.

A significant strength of this study is its large sample size, given the paucity of systematically-collected clinical data for PP, the low prevalence of the condition, and the aim for international diversity of the sample. Previous studies of PP phenotype had a cohort size of up to 130 [13] and included relatively homogenous national samples. The effort to recruit an international sample as well as participants from a wide range of healthcare settings and access increases the generalizability of our findings. A further strength of this study is that women who have experienced psychotic symptoms are recruited both with and without bipolar disorder diagnoses. For example, some previous research on postpartum psychosis has only included women with bipolar disorder [11, 14]. Inclusion of any individual who experienced a psychotic episode within 6 months of delivery, our data provides insight into a broader range of psychosis diagnoses, including Brief Psychotic Disorder and MDD with psychotic features, and we will explore the ability to identify phenomenological differences by factors such as time of onset.

There are important limitations to this study. Although the MINI-PDS is a gold-standard for structured clinical interviews examining psychotic and other psychiatric disorders, the reliability of collected data is limited by the time elapsed since the episode and the impact of psychosis on participant recall. In order to capture a broader sample, women are eligible to participate in the study at any point within the ten years following an episode of postpartum psychosis. Given this wide window, there is significant variation in the time elapsed between onset of episode and the study interview among the sample. For example, women whose episode took place many years ago may have flawed recall of their psychiatric symptoms and medication trials. Notably, poor insight and impaired or incomplete memory of psychotic episodes are common. Therefore, it is not unusual for study participants to report difficulty remembering the exact time of onset and presentation of psychotic symptoms; on such occasions, corroborating family or medical information is sought. The challenge inherent in symptom recall from the distant past is somewhat mitigated by the real temporal landmark (proximity to childbirth), and memorable nature associated with a postpartum psychotic episode. In addition, many women are hospitalized as part of treatment, history of which further consolidates the timeline of events. Further research on the presentation of PP should include assessment more proximate to the episode, medical records as a standard data source, and prospective study of women at risk for PP who are planning a pregnancy. Another limitation of the study may derive from our decision not to exclude women with histories of intermittent postpartum marijuana use (as long as such use was not associated with emerging psychotic symptoms) while excluding women with other frank substance use disorder. This qualification derived from our observation of the frequency of marijuana use among postpartum women and an effort not to limit the generalizability of our findings by excluding such potential subjects.

Despite these limitations, by working with invested stakeholders and patient advocates, investing in an online presence, and sharing the nature of this research on social media, an innovative recruitment strategy and subsequent engagement of a large and geographically diverse sample has been possible. Rather than recruit a group of subjects who derive from an exclusively inpatient setting, we aim to capture a broad range of experiences and clinical presentations, including women who never received inpatient hospitalization or women who live in areas with rural healthcare systems. In addition to geographic diversity, we also seek to recruit a representative population across racial, ethnic, and socioeconomic groups.

We will continue to engage with maternal mental health advocates and women with lived experience of PP to further identify participants that represent this broad range of experiences. MGHP3 gathers essential data on the clinical presentation of PP as well as genetic contributions, and the ongoing rigorous phenotyping and genetic sampling will contribute to our understanding of PP’s phenomenology. Modeled after existing psychiatric genetics initiatives [38, 39], MGHP3 is the first U.S.-based initiative in reproductive neuroscience to explore the phenomenology, timing of PP episode onset, symptom expression, and underlying psychiatric comorbidities with rigorous clinical evaluation in a large study sample. While previous genetic studies of psychiatric disorders have revealed inconsistent conclusions in terms of diagnostic classification in part due to study population heterogeneity, the broad range of MGHP3 recruitment platforms allows accession of a geographically and diagnostically diverse population of participants, permitting delineation of phenotypes in understudied populations using established, validated tools. Clarification of the heterogeneity of PP at the level of clinical symptom expression to genomic characteristics to neural circuitry can lead to a richer understanding of the disease which may have clinical implications with respect to specific pharmacologic treatment received, duration of treatment, and long-term prognosis.

## Supporting information

S1 Appendix Massachusetts General Hospital Postpartum Psychosis Questionnaire (MGHP3Q)^©^. (DOCX)
