# Supplementary material for: Establishment of the MGH Postpartum Psychosis Project: MGHP3
Source: PLoS One. 2023 Feb 9;18(2):e0281133. doi: 10.1371/journal.pone.0281133 (PMC9910633; doi:10.1371/journal.pone.0281133)
Supplement: S1 Appendix — (DOCX) [file pone.0281133.s001.docx]

**Massachusetts General Hospital Postpartum Psychosis Questionnaire (MGHP3Q)^©^** Version 3.0

*For each of the following items, record baseline information and information for the pregnancy preceding the index episode of postpartum psychosis. For women with more than one episode of postpartum psychosis, repeat starred questions for subsequent episodes.*

1. For how many pregnancies did you experience postpartum psychosis symptoms? (necessary to ask questions one pregnancy at a time): ______
2. Today’s date: __________________

**Demographics**

1. Date of Birth: _______________________
2. Age:______________________________
3. Height (inches): _____________________
4. Race:
   - American Indian or Alaska Native
   - Black or African American
   - White
   - Asian
   - Native Hawaiian or Other Pacific Islander
   - Other (specify)_______________
   - No primary race
   - Decline to answer
5. Ethnicity:
   - Hispanic or Latino
   - Non-Hispanic or Latino
   - Decline to answer
6. Legal marital status during pregnancy*:
   - Never Married
   - Married
   - Divorced
   - Widowed
   - Separated
   - Other (specify)______________
7. Current legal marital status:
   - Never Married
   - Married
   - Divorced
   - Widowed
   - Separated
   - Other (specify)______________
8. Health Insurance during pregnancy*:
   - Private/PPO/HMO/POS
   - Medicare
   - Medicaid
   - Another type of public insurance
   - Tricare
   - None
   - Other_____________________
9. Did this change in the year after you delivered? (Y/N)
   - If yes, to what?
   - Private/PPO/HMO/POS
   - Medicare
   - Medicaid
   - Another type of public insurance
   - Tricare
   - None
   - Other_____________________
10. Current Health Insurance:
    - Private/PPO/HMO/POS
    - Medicare
    - Medicaid
    - Another type of public insurance
    - Tricare
    - None
    - Other____________________
11. Education during pregnancy* (highest completed)
    - Less than high school
    - Some high school
    - High school diploma
    - Some college
    - Associate Degree
    - Bachelor’s Degree
    - Vocational or technical school
    - Postgraduate training
    - Other (specify)______________
12. Current Education: (highest completed)
    - Less than high school
    - Some high school
    - High school diploma
    - Some college
    - Associate Degree
    - Bachelor’s Degree
    - Vocational or technical school
    - Postgraduate training
    - Other (specify)______________
13. Employment during pregnancy*:
    - Employed for wages
    - Seeking work
    - Homemaker
    - Student
    - Disabled/unable to work
    - Retired
    - Other (specify)______________

**Medical comorbidities**

1. Have you ever had any of the following?* (Mark with an X)

| **Condition** | **Any time before pregnancy** | **During pregnancy*** | **After pregnancy up to 24 months** |
| --- | --- | --- | --- |
| High blood pressure |  |  |  |
| Pre-eclampsia, eclampsia, or HELLP syndrome; or postpartum pre-eclampsia |  |  |  |
| Diabetes |  |  |  |
| Toxoplasmosis |  |  |  |
| Obesity (BMI >30) |  |  |  |
| Hyperthyroidism |  |  |  |
| Hypothyroidism |  |  |  |
| Cancer |  |  |  |
| Autoimmune disease |  |  |  |
| Heart disease |  |  |  |
| Other chronic illness (specify): |  |  |  |

- *Check if no medical comorbidities above*

1. If yes to diabetes:

- Was it diagnosed as gestational diabetes?*

1. If yes to diabetes, was it treated with:*

- medication
- insulin
- other
- none

(If more than one period of diabetes with different treatments) Please describe:* _______ _______________________________________

1. (If hypo/hyperthyroidism during pregnancy) Associated with pregnancy?*

- Yes
- No

1. (If yes to cancer during pregnancy or postpartum) Did you receive chemotherapy?*

- Yes
- No

1. If more than one chronic illness, specify illness and periods:* ______________________________________

______________________________________

**General Obstetric History**

1. How many times have you been pregnant?
   - Number of live births: ______
   - TABs: ___________________
   - SABs: ___________________
   - Ectopic pregnancies: _______
   - Stillbirths: ________________
   - Infant deaths: ____________

Dates of birth of all children (indicate which led to episodes of postpartum psychosis with +): __

________________________________________

1. Biological sex of all children (in order of birth): ________________________________________ ________________________________________
2. Have you used assisted reproductive technology (ART)?
   - Yes
   - No
3. (If yes to ART) What type? __________________
4. For which pregnancy(ies)? __________________
5. History of hormonal contraceptive use?

| **Type of Contraception** | **Previous use (check if yes)** | **Ages of use** |
| --- | --- | --- |
| Hormonal IUD |  |  |
| Progesterone-only oral contraception |  |  |
| Estrogen and progesterone oral contraception |  |  |
| NuvaRing |  |  |
| Nexplanon/hormonal implant |  |  |
| Depo-Provera/ hormonal shot |  |  |

- *Check if no therapies above used*

1. Have you previously had hormone therapy?
   - Yes
   - No
2. (If yes to hormone therapy) What type? _______________________________________

**Past Psychiatric History and Treatment** (Verify diagnoses with Mini International Neuropsychiatric Interview (MINI). Inclusion/Exclusion dictated by MINI results.)

1. Have you ever been diagnosed with any psychiatric conditions other than postpartum psychosis?
   - Yes
   - No
2. Which psychiatric disorders have you been diagnosed with?

| **Diagnosis** | **Y/N** | **Age of symptom onset** | **Age of diagnosis** |  | **Diagnosis** | **Y/N** | **Age of symptom onset** | **Age of Diagnosis** |
| --- | --- | --- | --- | --- | --- | --- | --- | --- |
| Depression (MDD) |  |  |  |  | Attention Deficit Hyperactivity Disorder |  |  |  |
| Bipolar Disorder |  |  |  |  | Borderline Personality Disorder |  |  |  |
| Schizophrenia |  |  |  |  | Alcohol Use Disorder |  |  |  |
| Schizoaffective Disorder |  |  |  |  | Other Substance Use Disorder (specify): ____________________ |  |  |  |
| Psychotic Disorder (Not otherwise specified) |  |  |  |  | Eating Disorder |  |  |  |
| Anxiety Disorder |  |  |  |  | Post-Traumatic Stress Disorder |  |  |  |
| Panic Attacks |  |  |  |  | Other (specify): _______  ____________________ |  |  |  |
| Obsessive-Compulsive Disorder |  |  |  |  |  |  |  |  |

- *Check if no psychiatric diagnoses*

1. Did you have a psychiatric diagnosis prior to your episode of postpartum psychosis?
   - Yes
   - No
2. Have you ever been hospitalized for a psychiatric reason?
   - Yes
   - No
3. (If yes to 4) How many times have you been hospitalized for a psychiatric reason? ________
4. When were your hospitalizations? (calendar year) __________________________
5. (If yes to 4) Length of hospitalization (days):*___
6. Have you ever attempted suicide?
   - Yes
   - No
7. Number of suicide attempts: _______________
8. (If yes to 8) When? ______________________
9. Have you ever been to therapy?
   - Yes
   - No
10. (If yes) Type? ____________________________
11. How long were you in therapy, cumulatively? (months) _____
    - Check if ongoing
    - Check if in therapy just prior to postpartum psychosis episode
12. Have you used other non-medication treatments?
    - Exercise
    - Supplements
    - Meditation/mindfulness/yoga
    - Other: ___________________________
13. Did you ever have a psychotic episode before the postpartum period with experiences such as extreme paranoia, hearing or seeing special messages through the TV or radio, believing that you had special abilities or powers, or experiencing sensations, sounds, or images that other people around you couldn’t experience?
    - Yes
    - No

**Family History**

*The following questions are about mental health problems that currently or previously affected* ***your child’s*** *biological relatives. For each “siblings” category, mark the number of siblings with the disorder. For anyone with a positive history, mark hospitalizations with ^hsp^.*

| **Condition** | **Child** |  | **Maternal** |  |  |  |
| --- | --- | --- | --- | --- | --- | --- |
|  | **FS** | **HS** | **FS** | **HS** | **GM** | **GF** |
| Depression |  |  |  |  |  |  |
| Bipolar Disorder/ Mania |  |  |  |  |  |  |
| Anxiety Disorders |  |  |  |  |  |  |
| Obsessive Compulsive Disorder |  |  |  |  |  |  |
| Attention Deficit/ Hyper- activity Disorder |  |  |  |  |  |  |
| Autism Spectrum Disorder |  |  |  |  |  |  |
| Schizophrenia/ Other Psychotic disorder |  |  |  |  |  |  |
| Alcohol use disorder |  |  |  |  |  |  |
| Substance use disorder |  |  |  |  |  |  |
| Postpartum Psychosis |  |  |  |  |  |  |
| Postpartum Depression |  |  |  |  |  |  |
| Other: |  |  |  |  |  |  |
| Unknown/ unspecified |  |  |  |  |  |  |
| Suicide attempt |  |  |  |  |  |  |
| Completed suicide |  |  |  |  |  |  |

***FS = full sibling (specify number) HS = half sibling (specify number) GM = grandmother GF = grandfather**

Example:

| **Condition** | **Maternal** |  |  |  |  |
| --- | --- | --- | --- | --- | --- |
|  | **FS** | **HS** | **GM** | **GF** |  |
| Depression | 2^hsp (both)^ |  | X | X^hsp^ |  |

**Drug use**

1. Tobacco/cigarettes*

|  | None (0) | 1-10/day (<1/2 pack) | 11-20/day (1/2 – 1 pack) | >20/day (>1 pack) |
| --- | --- | --- | --- | --- |
| 12 mo before pregnancy |  |  |  |  |
| 1^st^ trimester |  |  |  |  |
| 2^nd^ trimester |  |  |  |  |
| 3^rd^ Trimester |  |  |  |  |
| < 24 mo after pregnancy |  |  |  |  |

1. If you changed your smoking habits during pregnancy, at what week did you change them?* _____________
2. Marijuana (smoking or other consumption)*

|  | None (0) | Not in a typical month | <once/ week | 1-3 times/ week | 4-6 times/ week | >6 times/ week | >once/day |
| --- | --- | --- | --- | --- | --- | --- | --- |
| 12 mo before pregnancy |  |  |  |  |  |  |  |
| 1^st^ trimester |  |  |  |  |  |  |  |
| 2^nd^ trimester |  |  |  |  |  |  |  |
| 3^rd^ trimester |  |  |  |  |  |  |  |
| <24 mo after pregnancy |  |  |  |  |  |  |  |

1. If you changed your habit during pregnancy, at what week did you change it?* ________________________
2. Alcohol: How many drinks did you have in a typical week?* (1 drink = 12-oz of beer/1 can/1 bottle; 8-oz malt liquor; 5-oz wine such that 1 glass of wine is equal to about 1/5 of a bottle of wine; 1.5-oz liquor/1 shot)

|  | None (0) | <1/week | 1-3/week | 4-6/week | >6/week | >once/day |
| --- | --- | --- | --- | --- | --- | --- |
| 12 mo before pregnancy |  |  |  |  |  |  |
| 1^st^ trimester |  |  |  |  |  |  |
| 2^nd^ trimester |  |  |  |  |  |  |
| 3^rd^ trimester |  |  |  |  |  |  |
| <24 mo after pregnancy |  |  |  |  |  |  |

1. How frequently did you binge drink?* (defined as 4 or more drinks within a 2-hour span for the average woman):

|  | Not in a typical month | Monthly | Weekly | 2-3 times/ week | >1/2 the days/week | Most days or every day in a typical month |
| --- | --- | --- | --- | --- | --- | --- |
| 12 mo before pregnancy |  |  |  |  |  |  |
| 1^st^ trimester |  |  |  |  |  |  |
| 2^nd^ trimester |  |  |  |  |  |  |
| 3^rd^ trimester |  |  |  |  |  |  |
| <24 mo after pregnancy |  |  |  |  |  |  |

1. How many days per week did you consume alcohol during an average month?*

|  | 0 days | 1-2 days | 3-4 days | 5-6 days | 7 days |
| --- | --- | --- | --- | --- | --- |
| 12 mo before pregnancy |  |  |  |  |  |
| 1^st^ trimester |  |  |  |  |  |
| 2^nd^ trimester |  |  |  |  |  |
| 3^rd^ trimester |  |  |  |  |  |
| <24 mo after pregnancy |  |  |  |  |  |

1. If you changed your habit during pregnancy, at what week did you change it?* ________________________
2. Were you using any prescription drugs in excess of their prescribed dose or frequency to get high, feel better, or for any reason other than their prescribed use?*

|  | Not in a typical month | <once/ week | 1-3/week | 4-6/week | >6/week | More than once/day |
| --- | --- | --- | --- | --- | --- | --- |
| 12 mo before pregnancy |  |  |  |  |  |  |
| 1^st^ trimester |  |  |  |  |  |  |
| 2^nd^ trimester |  |  |  |  |  |  |
| 3^rd^ trimester |  |  |  |  |  |  |
| <24 mo after pregnancy |  |  |  |  |  |  |

1. What medications or drugs were you using?* ___________________________________________________
2. If you changed your habit during pregnancy, at what week did you change it? *________________________
3. Did you use any illicit or street drugs, including cocaine, crack, opiates, heroin, speed, meth, or any others?*

|  | Not in a typical month | <once/ week | 1-3/week | 4-6/week | >6/week | More than once/day |
| --- | --- | --- | --- | --- | --- | --- |
| 12 mo before pregnancy |  |  |  |  |  |  |
| 1^st^ trimester |  |  |  |  |  |  |
| 2^nd^ trimester |  |  |  |  |  |  |
| 3^rd^ trimester |  |  |  |  |  |  |
| <24 mo after pregnancy |  |  |  |  |  |  |

1. What drugs were you using?* ________________________________________________________________
2. If you changed your habit during pregnancy, at what week did you change it?* ________________________

**Description of postpartum psychosis episode**

1. Date of delivery*:
2. Date of symptom onset:_
3. Symptom onset with respect to delivery (days)
4. Before we explore the specifics of your episode, it is helpful for us to get a broad overview of your experience, starting with how your symptoms came on, how they changed over time, what your family and friends might have noticed, and if you ended up receiving medical treatment, what prompted that? How would you describe the narrative of your postpartum psychosis?

________________________________________________________________________________________________________________________________________________________________________________________________________________________________________________________________________________________________________________________________________________________________________________________________________________________________________________________________________________________________________________________________________________________________________________________________________________________________________________________________________________________________________________________________________________________________________________________________________________________________________________________________________________________________________________________________________________________________________________________________________________________________________________________________________________________________________________________________________________________________________________________________________________________________________________________________________________________________________________________________________________________________________________________________________________________________________________________________________________________________________________________________________________________________________________________________________________________________________________________________________________________________________________________________________________________

1. On what date did your symptoms resolve? ___________________
2. Did you experience suicidal ideation during the episode?*
   1. Yes
   2. No
3. How long did the episode last?* (weeks) ______
4. Are the symptoms ongoing or resolved?
   1. Ongoing
   2. Resolved
5. Did the symptoms come and go (wax and wane) or were they consistent over time?*
   1. Wax and wane
   2. Consistent over time
6. Did your episode have elements of hyper-religiosity (or heavy religious themes)?*
   1. Yes
   2. No
7. Did you experience domestic violence proximate to or during your pregnancy or the postpartum period?*
   1. Yes, just prior to pregnancy
   2. Yes, during pregnancy
   3. Yes, postpartum
   4. No
8. Did you experience other types of trauma during your pregnancy or the postpartum period?*
   1. Yes, during pregnancy
   2. Yes, postpartum
   3. No
9. (If yes) Specify*: _________________________
10. How traumatic was this on a scale of 1-7, with 1 being not at all traumatic, and 7 being extremely traumatic?* ____________________
11. Were you hospitalized for your postpartum psychosis episode (partial or full)?*
    1. Yes
    2. No
12. If hospitalized, did you have access to your baby during your hospital stay?*
    1. Mother-baby inpatient unit
    2. Partial hospitalization
    3. Visiting hours with baby more than half of the days of the week
    4. Visiting hours with baby less than half of the days of the week
    5. No access to baby
13. What treatment did you receive? (use treatment tracking sheet)*
    1. Medication
    2. Therapy
    3. Natural supplements
    4. In-patient hospitalization
    5. Other ________________________
    6. Nothing
14. If you received medication, did you remain on it after the episode resolved?*
    1. Yes
    2. No
15. If not, did you relapse?*
    1. Yes
    2. No
16. How long did it take you to get well from the episode, including all psychiatric symptoms in the postpartum period?* (weeks) ___________
17. How traumatic was your postpartum psychosis episode on a scale of 1-7, with 1 being not at all traumatic and 7 being extremely traumatic?*__
18. At any point in your pregnancy or the postpartum period, was the Department of Children and Families or Child Protective Services involved?*
    1. Yes, an investigation was opened
    2. Yes, my child was taken away
    3. No
    4. Other (specify) __________________________________________________________
19. Has your postpartum psychosis episode shaped your decision on whether to have future children, or did it affect your plans to have subsequent children?*

- No, it has/did not shaped my decision
- It affected my decision a little
- It has been/was a moderate factor in my decision
- It has been/was a major or deciding factor in my decision
- Undecided/to be determined

1. How effective do you believe the immediate medical care you received was in treating your postpartum psychosis?* [field note: for example, this could be the care you received in the ER versus your long-term psychiatrist]
   1. N/A – Did not receive medical care
   2. Not at all effective or harmful
   3. A little effective or helpful
   4. Moderately effective or helpful
   5. Mostly effective or helpful
   6. Extremely effective or helpful
2. How effective do you believe the long-term medical care you received was in treating your postpartum psychosis?*

- N/A – Did not receive medical care
- Not at all effective or harmful
- A little effective or helpful
- Moderately effective or helpful
- Mostly effective or helpful
- Extremely effective or helpful

24. What do you think most contributed to your psychotic episode?*

- Lack of sleep
- Underlying illness
- Medical complication during pregnancy/postpartum
- Family history of psychiatric illness
- Other life stressors (specify) ____________
- I don’t know/unsure
- Other_______________________________

**Psychosocial support**

1. Did you receive paid maternity leave?*

- Yes
- No

1. How many weeks?* ______________________
2. Were you paid at your full salary?*
   - Yes
   - No
3. Did you take additional medical leave after your maternity leave ended due to your postpartum episode?*
   - Yes
   - No
4. How many weeks?*___
5. When did you go back to work with respect to your pregnancy (in weeks after delivery)?* ____ (N/A if you did not return to work)
6. Did you return at full time or part time?*
   - Full time
   - Part time
   - N/A
7. Who was available to support you and your baby during the postpartum?*
   - Family
   - Spouse or partner
   - Spouse’s family
   - Friends
   - Night nurse or other medical staff
   - Other (specify)_________________
8. Were family members present supportive/helpful overall?*
   - Not at all supportive/absent
   - A little supportive
   - Moderately supportive
   - Mostly supportive
   - Extremely supportive(If applicable)
9. Was your spouse or partner supportive?*
   - Not at all supportive/absent
   - A little supportive
   - Moderately supportive
   - Mostly supportive
   - Extremely supportive
10. Paternity leave offered?*
    - Yes
    - No
11. (If yes) How many weeks?* _______

**Specific Pregnancy History**

1. Was this pregnancy planned or unplanned?*
   - Planned
   - Unplanned
   - Unsure/Don’t remember
2. How far along were you in your pregnancy when you found out that you were pregnant?*
   - 0-4 weeks
   - 5-8 weeks
   - 9-12 weeks
   - 13-16 weeks
   - >16 weeks
3. Gestational age at delivery (weeks and days)* _______________________________________
4. Were you taking daily prenatal vitamins or folic acid supplements **before** you became pregnant?*
   - Yes
   - No
   - Unsure/Don’t Remember
5. (If yes) How long **before** you became pregnant were you taking vitamins or folic acid?*
   - <1 month
   - 1 month
   - 2 months
   - 3 months
   - >3 months
6. Did you take daily prenatal vitamins or folic acid supplements **while** you were pregnant?*
   - Yes
   - No
   - Unsure/don’t remember
7. (If yes), during what months of your pregnancy did you take a prenatal vitamin or multivitamin? (mark with X)*

| 1st | 2nd | 3rd | 4th | 5th | 6th | 7th | 8th | 9th |
| --- | --- | --- | --- | --- | --- | --- | --- | --- |
|  |  |  |  |  |  |  |  |  |

1. (Also if yes) During what months of your pregnancy did you take a separate folic acid (only) vitamin? (mark with X)*

| 1st | 2nd | 3rd | 4th | 5th | 6th | 7th | 8th | 9th |
| --- | --- | --- | --- | --- | --- | --- | --- | --- |
|  |  |  |  |  |  |  |  |  |

1. Were you aware of any fetal abnormalities during your pregnancy?*
   - Yes
   - No
   - Unsure/Don’t remember
2. (If yes) Specify abnormalities:* ___________________________________
3. During pregnancy, did you have any illness with a fever, cough, runny nose, diarrhea, achiness, marked fatigue, or other signs of infection?*
   - Yes
   - No
4. (If yes) What were your symptoms?* ________________________________________________________________________
5. (If yes) Did you receive medical care?* (specify or N/A) ______________________
6. (If yes) Did you take any medications?* (specify or N/A) ______________________
7. When did this occur during your pregnancy? (trimester)* _________________________
8. Did you live outside of the US for any part of your pregnancy?*
   - Yes
   - No
9. (If yes) Where?*_____________________
10. (If yes) When (trimester)?*______________
11. Delivery method:*
    - Vaginal
    - C-section
12. (If C-section) Was it emergent?*
    - Yes
    - No
13. Were you induced?
    - No
    - Yes, with Pitocin
    - Yes, my amniotic sac was ruptured (my water was broken)
    - Yes, my cervix was ripened with a prostaglandin (such as Cervidil, Cytotec)
14. Did you have any of the following:*
    - Pre-eclampsia
    - Eclampsia
    - HELLP syndrome
    - Hyperemesis gravidarum (severe nausea or vomiting)
    - Pelvic bone separation/symphysis pubis diastasis
    - Thyroid problems (hypo/hyper)
    - Other severe complications toward the end of pregnancy, during delivery, or immediately postpartum (specify) _____________________________________________________________________________
    - None or N/A
15. Did your baby get sent to the NICU?*
    - Yes
    - No
16. (If yes) How long was your baby in the NICU?*______________________________
17. Why was your baby sent to the NICU?* ____________________________________
18. Was your baby born premature?*
19. Has your baby had any serious health problems since delivery?* (specify or N/A) ________________________________________________________________________
20. Did you breastfeed?*
    - Yes
    - No
21. (If yes) For how long did you breastfeed?* _____________________________
    - Check here if ongoing
22. Was your decision to breastfeed, not to breastfeed, or to stop breastfeeding affected by your postpartum episode?*
    - No
    - Yes, I stopped breastfeeding because of the episode
    - Yes, I stopped breastfeeding because of the medications I was treated with for the episode
    - Yes, I started breastfeeding because of the episode
    - Yes, I did not breastfeed at all because of the episode
    - Yes, I did not breastfeed at all because of the medications I was treated with for the episode
    - I had already planned not to breastfeed
    - Other _______________________
    - N/A
23. Does your child have any ongoing medical problems?*
    - Asthma
    - Eczema
    - Recurrent infections
    - Allergies
    - Other _______________________
    - None to current knowledge
24. Does your child have any learning or cognitive deficits?*
    - Yes
    - No
    - Unknown/too early to know
25. Does your child have any psychiatric/behavioral problems?*
    - Yes
    - No
    - Unknown/too early to know
26. Does your child have autism?*
    - Yes
    - No
    - Unknown/Too early to know

Were all organic/medical causes for psychosis ruled out on the Mini International Neuropsychiatric Interview for Psychotic Disorder Studies?*

- Yes
- No (specify) _____________________
